# Do Molecular Profiles of Primary *Versus* Metastatic Radioiodine Refractory Differentiated Thyroid Cancer Differ?

**DOI:** 10.3389/fendo.2021.623182

**Published:** 2021-02-25

**Authors:** Cristiane J. Gomes-Lima, Leila Shobab, Di Wu, Dorina Ylli, Athanasios Bikas, Matthew McCoy, Rebecca Feldman, Wen Lee, Sarika N. Rao, Kirk Jensen, Vasily Vasko, Luiz Claudio Castro, Jacqueline Jonklaas, Leonard Wartofsky, Kenneth D. Burman

**Affiliations:** ^1^ Department of Internal Medicine, MedStar Clinical Research Center, MedStar Health Research Institute (MHRI), Washington, DC, United States; ^2^ Section of Endocrinology, MedStar Washington Hospital Center, Washington, DC, United States; ^3^ University of Brasilia School of Health Sciences, Postgraduate Program, Brasilia, Brazil; ^4^ Department of Internal Medicine, MedStar Georgetown University Hospital, Washington, DC, United States; ^5^ Innovation Center for Biomedical Informatics, Georgetown University Medical Center, Washington, DC, United States; ^6^ Caris Life Sciences, Medical Affairs, Phoenix, AZ, United States; ^7^ Division of Endocrinology, Mayo Clinic, Jacksonville, FL, United States; ^8^ Department of Pediatrics, Uniformed Services University of the Health Sciences, Bethesda, MD, United States; ^9^ Department of Pediatrics, University of Brasilia School of Medicine, Brasilia, Brazil; ^10^ Department of Medicine, Georgetown University, Washington, DC, United States

**Keywords:** differentiated thyroid cancer, metastases, radioiodine refractory, molecular profile, next generation sequencing

## Abstract

Management of metastatic radioiodine refractory differentiated thyroid cancer (DTC) can be a therapeutic challenge. Generally, little is known about the paired molecular profile of the primary tumor and the metastases and whether they harbor the same genetic abnormalities. The present study compared the molecular profile of paired tumor specimens (primary tumor/metastatic sites) from patients with radioiodine refractory DTC in order to gain insight into a possible basis for resistance to radioiodine. Twelve patients with radioiodine refractory metastases were studied; median age at diagnosis of 61 years (range, 25–82). Nine patients had papillary TC (PTC), one had follicular TC (FTC), and two had Hürthle cell TC (HTC). Distant metastases were present in the lungs (n = 10), bones (n = 4), and liver (n = 1). The molecular profiling of paired tumors was performed with a panel of 592 genes for Next Generation Sequencing, RNA-sequencing, and immunohistochemistry. Digital microfluidic PCR was used to investigate *TERT* promoter mutations. The genetic landscape of all paired sites comprised *BRAF*, *NRAS*, *HRAS*, *TP53*, *ATM*, *MUTYH*, *POLE*, and *NTRK* genes, including *BRAF* and *NTRK* fusions. *BRAF* V600E was the most common point mutation in the paired specimens (5/12). *TERT* promoter mutation C228T was detected in one case. PD-L1 expression at metastatic sites was highly positive (95%) for one patient with HTC. All specimens were stable for microsatellite instability testing, and the tumor mutation burden was low to intermediate. Therefore, the molecular profile of DTC primary and metastatic lesions can show heterogeneity, which may help explain some altered responses to therapeutic intervention.

## Introduction

Thyroid cancer is the fifth most frequent cause of malignancies among women with over 52,000 new cases of thyroid cancer estimated in 2020 among men and women in the US (1). Differentiated thyroid cancer (DTC) represents around 90% of all cases, and includes papillary thyroid cancer (PTC), follicular thyroid cancer (FTC), and Hürthle cell thyroid cancer (HTC) (2). Most cases have a good prognosis after appropriate treatment that involves surgical removal of the tumor and radioiodine (RAI) therapy in selected cases, especially in the presence of distant metastases (3). However, it is estimated that 6–20% of patients will develop distant metastasis that may impact the long-term outcomes of these individuals, particularly when the lesions become unresponsive to radioiodine (4–6).

The definition of RAI refractory DTC remains controversial. Several classifications have been proposed, including (a) malignant/metastatic tissue that does not concentrate RAI on a diagnostic radioiodine scan; (b) malignant tissue that does not concentrate RAI on a post-^131^I therapy scan; (c) tumor tissue that has lost the ability to concentrate RAI after previous evidence of radioiodine-avidity; (d) RAI that is concentrated in some lesions but not in others; (e) metastatic disease that progresses despite significant concentration of RAI; (f) after cumulative dosage of >600 mCi of ^131^I therapy (2, 7).

Management of metastatic radioiodine refractory DTC can be a therapeutic challenge. In contrast to metastatic disease in other non-thyroidal cancers, patients found to have metastatic DTC often have a slow progression of their disease, and management may consist of watchful waiting (3). Yet there is a proportion of these patients who will eventually have progression of their metastatic disease and will be candidates for targeted therapies (2, 8).

In recent years, with the advent of more comprehensive molecular panels and novel drugs targeting specific molecular abnormalities, application of precision medicine has provided a significant impact on the management of metastatic disease. This approach to management has become a reality for many types of advanced cancers, in which the best treatment option is chosen based on genetic features of the tumor (9). Effective treatment should consider the molecular nature of both the primary tumor and metastases, and a variety of techniques have been used to compare primary and metastatic tissues in different types of solid malignancies (10–15).

For thyroid cancer, however, little is known about the paired molecular profile of the primary tumor and its metastases, and if they harbor the same genetic abnormalities.

Only a few studies have addressed this question, employing different approaches and reaching diverse conclusions (16–19).

Absence of RAI avidity constitutes a major impediment to the delivery of effective therapy in patients with DTC. In this pilot study of patients with radioiodine refractory DTC, we used a comprehensive molecular panel available in clinical practice to compare the molecular profile of paired tumor specimens (primary tumor/metastatic sites). Moreover, we sought to investigate how genetic abnormalities found in our cohort interact with genes demonstrating enriched expression in the thyroid.

## Material and Methods

### Patient Selection

We performed a cross-sectional study including 12 patients with radioiodine (RAI) refractory metastases, selected from convenience sampling. Radioiodine refractoriness was defined as (a) disease having no RAI uptake at known sites of metastases on a post-^131^I therapy scan, or (b) tumor tissue that has lost the ability to concentrate RAI after previous evidence of radioiodine-avidity, or (c) metastatic disease that progresses despite significant concentration of RAI. We identified patients in current follow-up at MedStar Washington Hospital Center and MedStar Georgetown University Hospital whose tissues from the primary tumor and from one or more metastatic sites were available for molecular study. All specimens examined in this study were formalin-fixed paraffin-embedded (FFPE) samples.

### Next-Generation Sequencing

We used a pan-cancer panel of 592 genes from Caris^®^ Life Sciences to perform Next Generation Sequencing (NGS) of paired tumors—primary thyroid tumor and distant metastases—from each patient. This genetic panel includes the major thyroid cancer driver genes such as *BRAF, PTEN, RAS* and *RET.* The full list of genes is illustrated in the Supplementary Material (**Figure S1**). Caris^®^ Life Sciences is a CLIA-certified lab.

Extraction of DNA and RNA from FFPE t samples was performed using Qiagen extraction kit (QIAGEN, Hilden, Germany). Assessment of quality and quantity of nucleic acids prior to sequencing library was done with Agilent 4200 TapeStation System (Agilent Technologies, Santa Clara, CA, USA).

NGS was performed on genomic DNA isolated from FFPE tumor samples using the NextSeq platform (Illumina Inc., San Diego, CAS, USA). All variants were detected with >99% confidence based on allele frequency and amplicon coverage sequencing depth of coverage of >500 and with an analytic sensitivity of 5%. Variants detected were mapped to reference genome (hg19) and well-stablished bioinformatics tools were incorporated to perform variant calling functions. Genetic variants were categorized as “pathogenic”, “presumed pathogenic”, “variant of unknown significance”, “presumed benign” or “benign”, according to American College of Medical Genetics and Genomics (ACMG) standards.

RNA sequencing was performed on RNA isolated from FFPE tumor samples using the NextSeq platform (Illumina Inc., San Diego, CAS, USA) with whole transcriptome coverage.

Tumor mutation burden (TMB) was calculated based on the number of non-synonymous somatic mutations identified by NGS while excluding any known single nucleotide polymorphisms (SNPs). TMB is reported as mutations per megabase (Mb) sequenced.

Microsatellite instability (MSI) status was determined using NGS results. This method has been validated and does not require matched normal samples (20).

### PD-L1 Expression—Immunohistochemistry

The level of protein expression of PD-L1 in the paired tumor samples was determined with immunohistochemistry (IHC), performed on FFPE sections on glass slides using automated staining techniques. The procedures met the standards and requirements of the College of American Pathologists.

The primary antibody against PD-L1 was SP142 (Spring Bioscience, Pleasanton, CA, USA). An intensity score (0: no staining; 1+: weak staining; 2+: moderate staining; 3+: strong staining) and a proportion score to determine the percentage of cells staining positive (0–100%) were used. Conditions for a positive result included intensity ≥2+ and ≥5% of cells stained. Immunohistochemical results were evaluated by a board-certified pathologist.

### Microfluidic Digital PCR

Since Caris^®^ Life Sciences NGS panel used in this study could not detect *TERT* promoter abnormalities, we investigated mutations of *TERT* promoter in selected samples using microfluidic digital PCR, according to the following protocol:

- DNA Extraction
  Formalin-fixed paraffin-embedded (FFPE) tumor samples were deparaffinized with xylene and rehydrated through ethanol (100%). The samples were digested with Proteinase K, incubated at 55°C for 1 h with mild agitation and subsequently at 90°C for an additional hour. Extraction of DNA and RNA from FFPE cancer samples was performed using King Fisher Duo Thermo Fisher (Thermo Fisher Scientific Inc., Waltham, MA, USA) according to the manufacturer’s instruction. The extracted DNA and RNA elute was stored at −80°C. DNA and RNA quantification was performed by NanoDrop (Thermo Scientific, Inc., Waltham, MA, USA).
- Detection of *TERT* mutations using microfluidic digital PCR:
  3D Digital PCR analysis was performed on QuantStudio™ 3D Digital PCR System (Thermo Scientific, Inc., Waltham, MA, USA) using primers and probes for detection of *TERTC228T* and *TERTC250T* (Thermo Fisher Scientific). The final 14.5 μl of reaction mixture contained 8.7 μl QuantStudio™ 3D Digital PCR Master Mix, 0.43 μl of primer/probe mix, 10 ng of DNA. The mixture was loaded into the QuantStudio™ 3D Digital PCR Chip. For *TERT* promoter mutations, chips were run with the following conditions: one cycle at 50°C for 2 min; one cycle at 95°C for 10 min; 54 cycles at 55°C for 1 min, and 95°C for 15 s; one cycle at 60°C for 1 min. End point fluorescence data were collected on the QuantStudio™ 3D Digital PCR instrument and analyzed using the QuantStudio 3D AnalysisSuite software (Thermo Scientific, Inc., Waltham, MA, USA).
  The study was approved by Medstar Health Review Board.

### Bioinformatic Analysis

In order to explore the role of cancer driving mutations in the context of thyroid physiology, a functional association network was built using STRING (Search Tool for the Retrieval of Interacting Genes/Proteins) (21). Mutated genes identified in this cohort were combined with genes with enriched expression in the thyroid as reported by the Human Protein Atlas (22). The functional associations identified genes which connect a network associated with tissue specific molecular functions to cancer driving genes and provide clues to underlying mechanisms specific to thyroid cancer.

## Results

### Demographics and Clinical Aspects

A total of 12 patients (10 males) were included in this study. The median age at the time of diagnosis was 61 years (range, 25–82). Mean primary tumor size was 3.9 ± 2.2 cm SD; extra-thyroidal extension was present in 8/12 patients. Nine patients had papillary thyroid cancer (PTC), among which six were classic variant, one was follicular variant and one was columnar variant; one patient had follicular thyroid cancer (FTC) and two had Hürthle cell thyroid cancer (HTC). Distant metastases were present in the lungs (n = 10), bones (n = 5), and liver (n = 1). Biopsy from metastatic sites were taken at different points, according to clinical indication. Subjects # 4, 9, 11, and 12 were diagnosed with distant metastases and had the biopsy of the metastatic lesion before the thyroidectomy. According to the definition of RAI-refractoriness, three patients had no RAI uptake at known sites of metastases on a post-^131^I therapy scan (subjects # 4,11, 12), four patients had lesions that have lost the ability to concentrate RAI after previous evidence of radioiodine-avidity (subjects # 3, 7, 8, 9), and five patients had metastatic disease that has progressed despite significant concentration of RAI (subjects # 1, 2, 5, 6, 10). The baseline features of this cohort are described on **Table 1**.

**Table 1:** Baseline characteristics.

	N = 12
Gender	
Male	10
Female	2
Age at diagnosis	
Mean	61
SD	15.9
Median	61
Range	25–82
Thyroid tumor (cm)	3.9 ± 2.2
Histology	
PTC - classic	6
PTC – follicular variant	2
PTC – columnar variant	1
FTC	1
HTC	2
Extrathyroidal extension	8
Metastases at diagnosis	9
Distribution of metastasis	
Neck LN	11
Mediastinal LN	3
Lung	10
Bone	5
Atypical (liver)	1

PTC, papillary thyroid cancer; FTC, follicular thyroid cancer; HTC, Hürthle cell thyroid cancer; LN, lymph node.

### Next-Generation Sequencing

**Figure 1:**
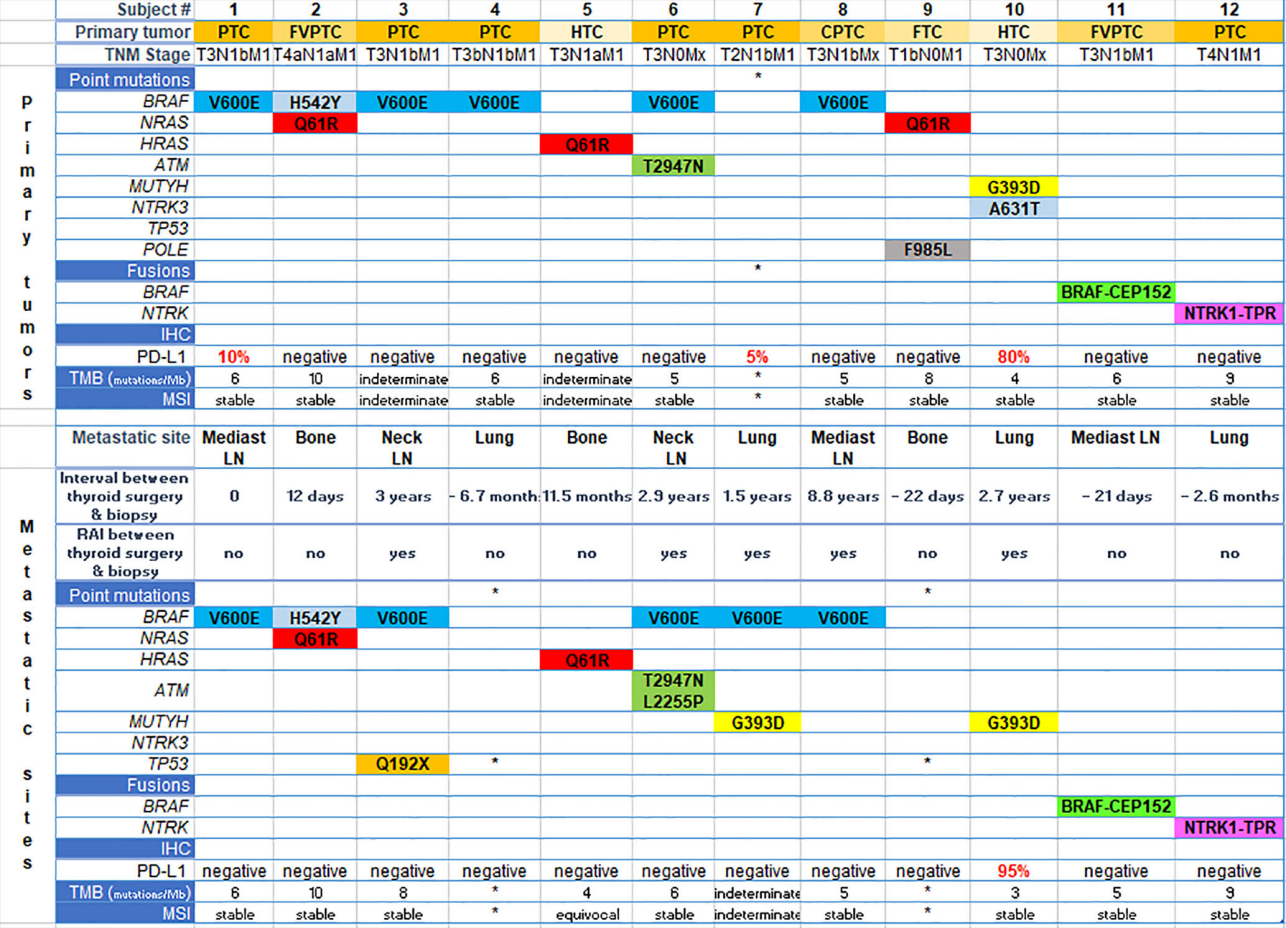
Genetic landscape and immunohistochemistry (PD-L1 expression) of paired sites. Each column represents one subject. The upper part of the figure refers to aspects of primary tumors and the bottom, to metastatic sites specified in the figure. *means quality or quantity not sufficient for analysis. PTC, papillary thyroid cancer; FVPTC, follicular variant of papillary thyroid cancer; CPTC, columnar variant of papillary thyroid cancer; FTC, follicular thyroid cancer; HTC, Hürthle cell thyroid cancer; IHC, immunohistochemistry; TMB, tumor mutation burden; Mb, megabase; MSI, microsatellite instability; RAI, radioiodine; LN, lymph node.

The genetic landscape of all paired sites comprised *BRAF, NRAS, HRAS, TP53, ATM, MUTYH, POLE* and *NTRK* genes, including *BRAF* and *NTRK* fusions (**Figure 1** and **Table 2**). Out of 12 patients, five presented with *BRAF* V600E mutation in both the primary and metastatic tumors. One patient had concomitant *BRAF* H542Y and *NRAS* Q61R mutations. Driver mutations were consistent between the sites, but in three patients other single nucleotide variants were also present, either solely in the metastatic tumor (subjects #3 and #6) or in the primary tumor (subject #10). Two patients showed gene fusions in both sites: *BRAF-CEP152* (#11) and *NTRK1-TPR* (#12). Comparison between the sites was not possible in three patients due to technical reasons, which was lack of quality or quantity of DNA for analysis.

**Table 2:** Genetic landscape and immunohistochemistry.

	Primary	Metastases
**Point mutations**		
* BRAF* V600E	5	5
* BRAF* H542Y	1	1
* NRAS* Q61R	1	1
* TP53* Q192X	0	1
* ATM* T2947N	1	1
* ATM* L2255P	0	1
* MUTYH* G393D	1	2
* NTRK3* A631T	1	0
**Gene fusions**		
* BRAF-CEP152*	1	1
* NTRK1-TPR*	1	1
**Immunohistochemistry (IHC)**		
Positive PD-L1 expression	3/12	1/12
**Tumor Mutational Burden (TMB)**	4 -10/Mb	3 -10/Mb
**Microsatellite Instability (MSI)**	Stable	Stable

Mb, Megabase.

Nine specimens were stable for microsatellite instability testing (MSI) in primary tumors, and eight in the metastatic specimens (**Figure 1** and **Table 2**).

Tumor mutation burden (TMB) ranged from four to 10 mutations/megabase in primary tumors, and from three to 10 mutations/megabase in metastatic specimens. This aspect could not be determined in three cases from primary and metastatic sites (**Figure 1** and **Table 2**).

### PD-L1 Expression—Immunohistochemistry

In the primary tumor, PD-L1 expression was positive in two patients with PTC (5 and 10%) and in one patient with HTC (80%), while in the metastatic tumor only the latter patient remained positive (95%) (**Figure 1** and **Table 2**).

### Microfluidic Digital PCR

*TERT228* and *TERT250* were tested in tissue samples from subjects # 1, 2, 4, 6, 8, 9, 11, and 12 due to limited availability of specimens (**Table 3**).

**Table 3:** Results from microfluidic digital PCR.

Subject # and site tested	*TERT* promoter	
	C228	C250
#1—thyroid	ND	ND
#2—bone	C228T	ND
#4—thyroid	ND	ND
#6—thyroid	ND	ND
#8—mediastinal LN	ND	ND
#9—thyroid	ND	ND
#11—thyroid	ND	ND
#12—thyroid	ND	ND

LN, lymph node; ND, not detected.

*TERT* promoter mutation C228T was detected in subject # 2.

*TERT250* mutation was not detected in any of the examined cases.

### Bioinformatics Analysis

**Figure 2:**
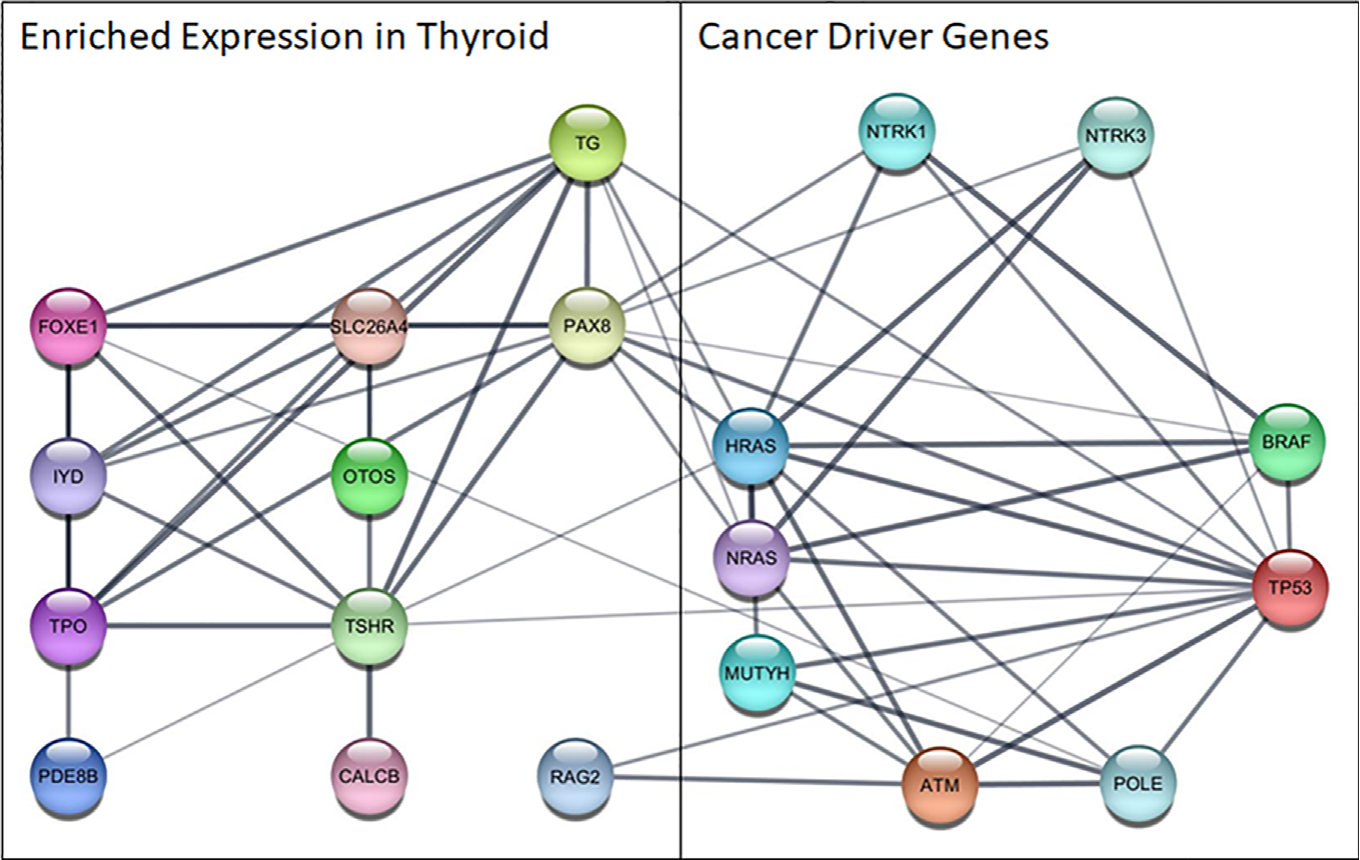
Functional network among cancer driver genes identified in this cohort and genes highly expressed in the thyroid. Nodes represent proteins, edges show functional associations and line thickness corresponds to the strength of the supporting evidence. ATM, ATM serine/threonine kinase; BRAF, B-Raf proto-oncogene,serine/threonine kinase; CALCB, Calcitonin Related Polypeptide Beta; FOXE1, Forkhead Box E1; HRAS, HRas Proto-Oncogene, GTPase; IYD, Iodotyrosine Deiodinase; MUTYH, MutY DNA Glycosylase; NRAS, NRAS Proto-Oncogene, GTPase; NTR, Nerve Growth Factor Receptor; NTRK1, Neurotrophic Receptor Tyrosine Kinase 1; NTRK3, Neurotrophic Receptor Tyrosine Kinase 3; OTOS, Otospiralin; PAX8, Paired Box 8; PDE8B, Phosphodiesterase 8B; POLE, DNA Polymerase Epsilon, Catalytic Subunit; RAG2, Recombination Activating 2; SLC26A4, Solute Carrier Family 26 Member 4; TG, Thyroglobulin; TP53, Tumor Protein P53; TPO, Thyroid Peroxidase; TSHR, Thyroid Stimulating Hormone Receptor.

The bioinformatics analysis revealed several interesting functional associations between the cancer and thyroid gene sets (**Figure 2**). The edges that link the proteins identified through the molecular profiling to these thyroid specific genes represent the results of several bioinformatic functional analysis workflows. The links between these two gene sets largely represent the results of text mining associations, where the two genes are explicitly mentioned in the same peer reviewed publication. There was also some supporting evidence of interactions between homologs in other species. The analysis revealed several key associations between highly expressed genes in the thyroid and those that drive thyroid cancer. Of particular note are PAX8, a transcription factor that modulates expression of the sodium iodide symporter, and RAG2, which mediates DNA cleavage during V(D)J recombination, and their associations with TP53.

## Discussion

This study highlights the differences and similarities between the molecular nature of the primary tumor and a metastatic site in patients with RAI refractory DTC. Although the overall mutational status was similar, some differences were identified that could have therapeutic impact in the context of precision medicine.

In spite of the higher incidence of thyroid cancer in women, in this cohort of advanced thyroid cancer, 10/12 subjects were men. This is consistent with data showing that men with DTC are more likely to present with more advanced disease, aggressive histological subtypes, and older age (23).

Consistently with previously published data (24, 25), *BRAF* point mutation was the most common genetic abnormality observed in this cohort, present in seven out of 12 patients (58%). Among these patients, only one had a non-V600 *BRAF* mutation. This patient (subject #2) presented a *BRAF* H542Y mutation concomitant with an *NRAS* Q61R mutation in both the primary and the metastatic tumor in bone lesion. According to COSMIC v91 (26, 27), this missense mutation has been described in colon cancer and melanoma, while the TCGA database does not report it (28, 29). *BRAF* and *RAS* mutations are known to be mutually exclusive, but simultaneous mutations have been described in colorectal cancer (30). The overall impact of simultaneous *BRAF* and *RAS* mutations remains unclear in the setting of thyroid cancer. However, it is believed that *BRAF* non-V600 mutations are less responsive to the RAF inhibitors, vemurafenib and dabrafenib, which could narrow the therapeutic options for these tumors (31). Moreover, this subject also presented with a *TERT* promoter mutation C228T in the bone specimen which is in accordance with the clinical picture of an aggressive tumor with local and distant metastases. The current version of Caris^®^ Life Sciences tumor profiling employs whole exome sequencing instead of next-generation sequencing, which allows the detection of *TERT* promoter mutations.

Among all kinases, *BRAF* fusions are the most commonly observed across several tumor types, including prostate cancer, melanoma, radiation-induced thyroid cancer, and pediatric low-grade gliomas. However, the full spectrum of *BRAF* fusions in human cancer remains incomplete (9, 32). In this cohort we describe a novel *BRAF* fusion: *BRAF-CEP152*.

Additionally, we report the occurrence of *NTRK1* fusion with a known partner, *TPR*. This partner harbors a dimerization domain that potentially activates the downstream TRK kinase domain (33). *TRK* fusions have been implicated in the pathogenesis of congenital fibrosarcoma, secretory breast carcinoma, and papillary thyroid cancer (32). These rare fusions have been reported in only 1% of advanced PTCs (25), yet they are targetable. Larotrectinib, a highly selective inhibitor of the three TRK proteins, is FDA-approved for solid tumors harboring fusion in these genes (34).

Six out of 12 patients (50%) showed the same genetic abnormalities between the primary and the metastatic tumor. In three cases the comparison was not possible due to insufficient quality or quantity of DNA or RNA for analysis, which highlights the importance of tissue sampling in the current era of molecular studies. Two patients had new point mutations in the metastatic tumor: subject # 3 had a *TP53* mutation and subject #6 had a second hit on *ATM* gene. These are both DNA repair genes that, once mutated, could provide survival advantage to the tumor. On the other hand, one subject (#10) had a *NTRK3* missense mutation in the primary tumor that was not seen in the metastatic lesion. This could imply that the mutation was not providing any advantage to the tumor and was selected out. These patients had been previously treated with at least one dose of radioiodine in the interval between thyroidectomy and the biopsy of the metastatic tumor. None of them have received external radiation in this interval.

The immune landscape of papillary thyroid cancer is characterized as an inflammatory subtype, with low to moderate tumor cell proliferation and lower levels of somatic copy number alterations than other subtypes (35). PTCs are tumors with low mutational burden due to low neoantigen expression, which leads to poor immunogenicity (36). A comprehensive screening for PD-L1 expression in thyroid cancer found that only 6.6% of cases expressed PD-L1 at 1% threshold, with higher positivity in follicular and anaplastic carcinomas (37). In our cohort, two patients with PTC expressed PD-L1 in the primary tumor but not in the metastatic sites, a mediastinal lymph node and a lung nodule. In addition, one patient with Hürthle cell thyroid cancer (HTC) had very high PD-L1 expression in both the thyroid tumor and in the specimen from lung biopsy. This finding may have direct therapeutic applications. Our samples also showed an overall low mutational burden (≤10 mutations/megabase) in matched specimens.

Previous studies have addressed genetic abnormalities in primary thyroid cancer and matched metastases. Sohn et al. used a next-generation sequencing panel of 50 genes and found highly concordant genetic abnormalities between primary and metastatic specimens from 17 DTC patients, including 10 patients with PTC and seven patients with FTC (17). In another study, Masoodi et al. used whole-exome sequencing to compare 14 trios of matched distant metastasis, primary PTC tumors and normal samples. Those authors found striking heterogeneity between the primary PTC tumors and metastatic specimens, attributed mainly to passenger mutations (19). In comparison to those studies, our study population is more heterogeneous and reflects real life practice in a tertiary hospital. Additionally, the genetic diversity that we found is also noteworthy, despite the small sample size. Our data emphasizes the importance of identifying the molecular profile of radioiodine refractory metastatic lesions in the era of precision medicine. The identification of a targetable mutation or fusion will definitely influence the therapeutic decision.

The disrupted biological process that results in tumor growth and metastasis occurs within the context of gene expression patterns of the diseased tissue, and may result in uniquely targetable interactions that drive subtypes of cancer in a particular tissue. The functional associations identified between highly expressed genes in the thyroid and those found to drive cancer in patients with metastatic thyroid cancer are helpful in generating novel hypotheses about cancer driving mechanisms unique to thyroid cancer. *RAG2* is regulated by *TP53* derived peptides in functional experiments investigating DNA repair mechanisms (38, 39), and lower expression of PAX8 resulted from mutated *TP53* in thyroid epithelial cells (40). Additionally, functional associations between RAG2 and ATM were found to cooperatively facilitate repair of double-strand breaks (41) and multiple functional interactions were found between PAX8 and the cancer genes in homologs from other species in the Biological General Repository for Interaction Datasets (BioGRID) (42). Further investigation into the functional relationships of RAG2 and PAX8 with cancer associated genes, especially those that are not typically expressed in the thyroid, is needed to fully understand and potentially disrupt these disease pathways using innovative therapeutic interventions.

Limitations in the interpretation of our data include the relatively small sample size and the analysis of only one metastatic lesion in each patient. Additionally, specimens were taken at different times during the patients’ follow-up, with RAI therapy administered between the collection of some samples. Nevertheless, the data and conclusions drawn provide new and useful information and reflect real life practice.

## Conclusion

In this study, we demonstrate that molecular abnormalities in the primary tumor and metastatic sites of DTC can show heterogeneity. Driver mutations remained essentially the same but new mutations were observed in some cases. A better understanding of the genetic and biologic aspects of metastatic DTC is expected to contribute to the development of more effective targeted therapies.

## Author’s Note

The content of this manuscript has been presented in part at the 89^th^ Annual Meeting of the American Thyroid Association, Chicago, IL, 2019 as a Highlighted Oral Presentation (43).

## Data Availability Statement

The datasets presented in this study can be found in online repositories. The names of the repository/repositories and accession number(s) can be found below: https://www.ncbi.nlm.nih.gov/bioproject/692614

## Ethics Statement

The studies involving human participants were reviewed and approved by the Medstar Health Review Board. The patients/participants provided their written informed consent to participate in this study.

## Author Contributions

Conceptualization: CG-L, LS, and KB. Methodology: CG-L, LS, DW, DY, RF, AB, VV, and KJ. Resources: VV and KJ. Pathology review: WL. Bioinformatic analysis: MM. Writing—original draft preparation: CG-L, LC, and KB. Writing—review and editing: LS, DW, DY, MM, SR, LC, VV, JJ, LW, and KB. Supervision: CG-L, LW, and KB. Project administration: CG-L, DY, and KB. Funding acquisition: LW and KB. All authors contributed to the article and approved the submitted version.

## Funding

This work was supported by The Catherine Heron and Al Schneider Fellowship in Thyroid Cancer.

## Conflict of Interest

RF is employed by Caris Life Sciences.

The remaining authors declare that the research was conducted in the absence of any commercial or financial relationships that could be construed as a potential conflict of interest.

## Supplementary Material

Supplementary Figure 1 – Caris^®^ Life Sciences NGS gene list.
