# Supplementary material for: Do Molecular Profiles of Primary Versus Metastatic Radioiodine Refractory Differentiated Thyroid Cancer Differ?
Source: Front Endocrinol (Lausanne). 2021 Feb 25;12:623182. doi: 10.3389/fendo.2021.623182 (PMC7949910; doi:10.3389/fendo.2021.623182)
Supplement: Supplementary Figure 1 — – Caris® Life Sciences NGS gene list. [file DataSheet_1.pdf]

Next-Generation Sequencing Gene List

Whole Exome Sequencing – Genomic Stability Testing (DNA)	
Microsatellite Instability (MSI)	Tumor Mutational Burden (TMB)*

Whole Exome Sequencing – Genes most commonly associated with cancer below.

Point Mutations and Indels (DNA)

ABL1	BCL2	EPHA2	GLI2	KDM6A	MPL	PARP1	RABL3	SOCS1
AIP	BCOR	FANCB	GNA11	KDR	MSH3	PHOX2B	RAD51C	SPOP
AKT1	BTK	FANCF	HIST1H3B	LYN	MST1R	PIK3CB	RAD51D	SRC
AMER1	CD79B	FANCI	HIST1H3C	LZTR1	MUTYH	PMS1	RHOA	TERT
AR	CDH1	FANCM	HNF1A	MAPK1	NBN	POLD1	SDHA	TMEM127
ARAF	CDK12	FAT1	HOXB13	MAPK3	NOTCH1	PPP2R1A	SDHAF2	VHL
ATRX	CXCR4	FOXL2	HRAS	MAX	NRAS	PRKACA	SETD2	XRCC1
B2M	DNMT3A	FYN	KDM5C	MED12	NTHL1	PRKDC	SMARCA4	YES1

Point Mutations, Indels and Copy Number Alternations* (DNA)

ALK	BRIP1	CSF1R	FANCD2	FUBP1	KMT2A	MSH2	PBRM1	RAD50	SMO
APC	CARD11	CTNNB1	FANCE	GATA3	KMT2C	MSH6	PDGFRA	RAF1	SPEN
ARID1A	CBFB	CYLD	FANCG	GNA13	KMT2D	MTOR	PDGFRB	RB1	STAT3
ARID2	CCND1	DDR2	FANCL	GNAQ	KRAS	MYCN	PIK3CA	RET	STK11
ASXL1	CCND2	DICER1	FAS	GNAS	LCK	MYD88	PIK3R1	RNF43	SUFU
ATM	CCND3	EGFR	FBXW7	H3F3A	MAP2K1	NF1	PIM1	ROS1	TNFAIP3
ATR	CDC73	EP300	FGFR1	H3F3B	MAP2K2	NF2	PMS2	RUNX1	TNFRSF14
BAP1	CDK4	ERBB2	FGFR2	IDH1	MAP2K4	NFE2L2	POLE	SDHB	TP53
BARD1	CDK6	ERBB3	FGFR3	IDH2	MAP3K1	NFKBIA	POT1	SDHC	TSC1
BCL9	CDKN1B	ERBB4	FGFR4	IRF4	MEF2B	NPM1	PPARG	SDHD	TSC2
BLM	CDKN2A	ERCC2	FH	JAK1	MEN1	NSD1	PRDM1	SF3B1	U2AF1
BMPR1A	CHEK1	ESR1	FLCN	JAK2	MET	NTRK1	PRKAR1A	SMAD2	WRN
BRAF	CHEK2	EZH2	FLT1	JAK3	MITF	NTRK2	PTCH1	SMAD4	WT1
BRCA1	CIC	FANCA	FLT3	KEAP1	MLH1	NTRK3	PTEN	SMARCB1	
BRCA2	CREBBP	FANCC	FLT4	KIT	MRE11	PALB2	PTPN11	SMARCE1	

Whole Transcriptome Sequencing – Genes most commonly associated with cancer listed below.

Fusions (RNA)

ABL	BRD3	FGFR3	INSR	MYB	NUMBL	PRKCA	RSPO3
AKT3	BRD4	ERG	MAML2	NOTCH1	NUTM1	PRKCB	TERT
ALK	EGFR	ESR1	MAST1	NOTCH2	PDGFRA	RAF1	TFE3
ARHGAP26	EWSR1	ETV1	MAST2	NRG1	PDGFRB	RELA	TFEB
AXL	FGR	ETV4	MET	NTRK1	PIK3CA	RET	THADA
BCR	FGFR1	ETV5	MSMB	NTRK2	PKN1	ROS1	TMPRSS2
BRAF	FGFR2	ETV6	MUSK	NTRK3	PPARG	RSPO2	

Variant Transcripts (RNA)

Variant Transcripts (RNA)
AR-V7
EGFR vIII
MET Exon 14 Skipping
